# Analysis of Four Solvatomorphs of Betulin by TG–DTA–EI/PI–MS System Equipped with the Skimmer-Type Interface

**DOI:** 10.1007/s13659-020-00243-3

**Published:** 2020-05-15

**Authors:** Peng-Hui Yuan, Yan-Cai Bi, Bin Su, De-Zhi Yang, Ning-Bo Gong, Li Zhang, Yang Lu, Guan-Hua Du

**Affiliations:** 1 Beijing City Key Laboratory of Polymorphic Drugs, Center of Pharmaceutical Polymorphs, Institute of Materia Medica, Chinese Academy of Medical Sciences and Peking Union Medical College, Beijing, 100050 People’s Republic of China; 2 Soteria Pharmaceutical Co., Ltd, Laiwu, 271100 People’s Republic of China; 3 Beijing City Key Laboratory of Drug Target and Screening Research, National Center for Pharmaceutical Screening, Institute of Materia Medica, Chinese Academy of Medical Sciences and Peking Union Medical College, Beijing, 100050 People’s Republic of China

**Keywords:** Betulin, Solvatomorphs, TG–MS, PI method

## Abstract

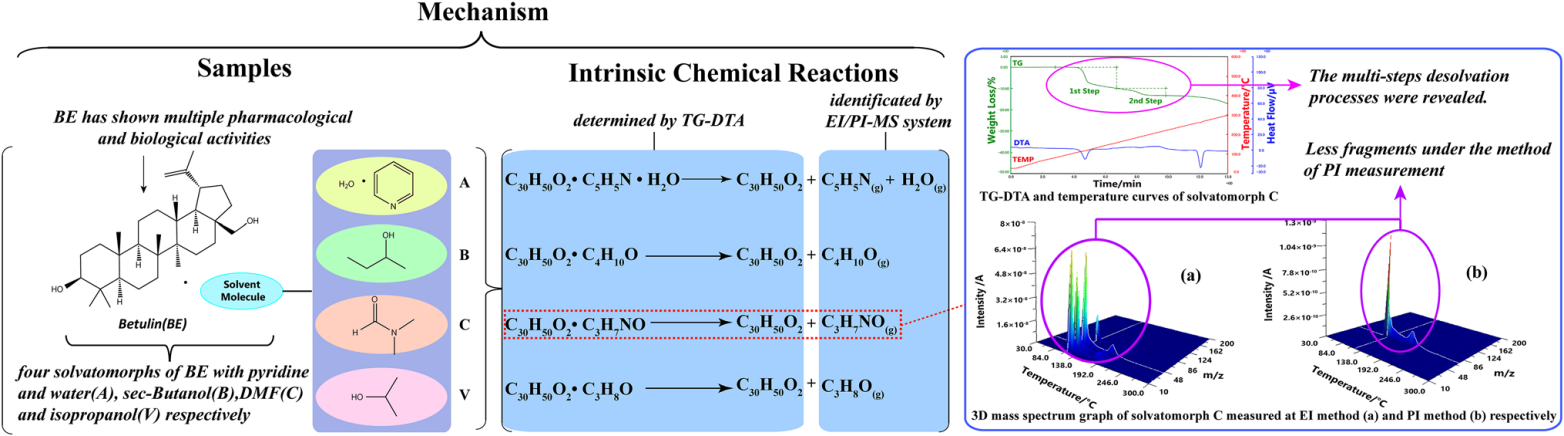

## Introduction

Betulin [BE, PubChem CID: 72326; lup-20 (29)-ene-3β, 28-diol], is a natural lupine-type pentacyclic triterpenoid, which is extracted and isolated mainly from the bark of birch trees (*Betula* spp., Betulaceae). BE can also be isolated from the leaves of *Syzygium formosanum* or those of *Diospyros leucomelas* [1, 2]. BE has exhibited a wide range of pharmacological and biological activities. As a great potential natural product, BE not only has anti-HIV, anti-microbial, anti-malarial, and anti-inflammatory properties, but it also processes multiple anti-cancer pharmacological activities. In terms of anti-viral and anti-cancer effects, BE is considered a potential therapeutic agent for treating several important viral infections via different host–virus interaction pathways, and research is currently being conducted on improving the apoptotic effects or targeted delivery of BE to cancer cells [3]. Besides, anti-fibrotic activity is another significant property of BE [3, 4]. Recent research has also indicated that BE modulates the differentiation of osteoclasts and thus has become an effective structural scaffold for developing therapeutic agents that can treat osteoclast-mediated bone disorder diseases, such as osteoporosis [5].

Solvatomorphism, or pseudopolymorphism, refers to crystal systems with different crystal structures of the same substance combined with different amounts or types of solvent molecules. Compared with solvatomorphs, polymorphs are substances with different unit cells consisting of the same elemental compositions [6]. Briefly, due to the introduction of solvent molecules, solvatomorphs usually show higher thermodynamic instability than polymorphs. Solvatomorphs can convert into polymorphs after implementing certain approaches, such as the vacuum drying method. In general, utilizing various organic solvents in the extraction, isolation or purification processes of natural products is unavoidable; thus, some innovative techniques have been developed, for example ultrasound-assisted extraction techniques, to reduce the amount of used solvents [7]. Certain natural products can easily form solvatomorphs with solvents in the extraction, isolation, or purification processes because of their inherent molecular structural properties. In some cases, solvatomorphs is regarded the only kind of crystalline form of natural products utilizable in single-crystal X-ray diffraction (SXRD) experiments, which is the most authoritative structural confirmation method besides total synthesis. BE, due to its all-crystalline form reportedly in the form of solvates, is a good example for meeting the experimental requirement [8, 9]. The presence of organic solvents will increase the toxicity of pharmaceutics, but solvatomorphs still have potential for being selected as the form for clinical use because of their good solubility or stability. For instance, cabazitaxel, as an anti-tumor drug, was approved in the form of a solvate with acetone by the Food and Drug Administration. Therefore, solvatomorph is valuable and significant in solid state with regard to natural products.

However, obtaining suitable single crystals of solvatomorphs for SXRD analysis is not easy. Highly specific analysis techniques need to be carried out and used to analyze the solvent components accurately in the solvate crystals of natural products. The normal thermogravimetry (TG)–mass spectrometry (MS) coupling analysis system has been considered a powerful hyphenated technique for evolved gas analysis [10]. TG–MS is widely used in chemical engineering, energy industry, and thermophysics research, especially in analyzing the pyrolysis processes of polymer, coal, and various inorganic substances, and the research of their dynamic reaction processes [11–13]. As a pharmaceutical solid-state analytical technique, TG–MS analysis can obtain results that provide more abundant information about thermodynamic reaction processes than other characterization methods, such as powder X-ray diffraction, differential scanning calorimetry, Fourier transform infrared, and Raman spectroscopy, which are used to identify and characterize solvatomorphs. Compared with the SXRD method, TG–MS analysis provides more flexible requirements for the samples, particularly those solvatomorphs that hardly form as single crystals, which are suited for SXRD experiments. Previous researchers have also reported that the TG–MS technique is a feasible and reliable method for analyzing and characterizing solvates [14–17]. However, problems during measurements, such as re-condensation of evolved volatile gases and excessive fragments causing the overlay of characteristic peaks in mass spectrometry, have still not been solved successfully. Past researchers have also shown that the skimmer sampling interface of TG–MS can effectively solve the recrystallization problem of samples [18]. In this study, the TG–differential thermal analysis (DTA)–electron ionization (EI)/photoionization (PI)–MS system equipped with the skimmer-type interface was employed owing to its advantages in dealing with the abovementioned problems.

Four different solvatomorphs of BE and their single-crystal structures were reported by Yang et al*.* [8, 9] in a previous research. However, their thermodynamic reaction processes had not been studied further. In the present study, crystal growth experiments using various organic solvents were designed to obtain the single crystals of the four solvatomorphs, in which the ratios of BE were 1:1:1 to pyridine and water (A), 1:1 to *sec*-butanol (B), 1:1 to *n,n*-dimethylformamide (DMF) (C), and 1:1 to isopropanol (V), following the methods of former authors. The identification and characterization of the four solvatomorphs of BE by using the TG–DTA–EI/PI–MS system was accomplished for the first time in this study. The system was employed to investigate the different evolving behaviors of volatile gas in the four solvatomorphs of BE. Additionally, the relationships between desolvation behavior and intermolecular interaction in the solvatomorphs of BE and the differences between EI and PI for analyzing solvatomorphs were revealed.

## Results and Discussion

### Identification of Evolved Volatile Gases of the Four Solvatomorphs of BE

The evolved volatile gases of the four solvatomorphs of BE were confirmed clearly by the TG–DTA–EI/PI–MS system. The identification of the chemical structures of the evolved gases via PI measurements is listed in Table 1. Table 2 shows the total analysis results.

**Table 1:** Identification of the chemical structure of the evolved gases of four solvatomorphs of BE at PI method

Solvatomorphs	m/z	Formula	Name	Chemical structure	Formula of the neutral loss moiety
Solvatomorph A	79	C_5_H_5_N^+^	Pyridine		–
Solvatomorph B	74	C_4_H_10_O^+^	*sec*-Butanol		–
	56	C_4_H_8_^+^	1-Butene		H_2_O
Solvatomorph C	73	C_3_H_7_NO^+^	DMF		–
Solvatomorph V	60	C_3_H_8_O^+^	Isopropanol		–
	45	C_2_H_5_O^+^	Isohydroxyethyl		CH_3_•
	44	C_2_H_4_O^+^	Ethenol		CH_4_

**Table 2:** Evolved gases analysis results of four solvatomorphs by EI and PI method

Solvatomorphs	EI (m/z)	PI (m/z)	Evolved gas	Ionization energy (eV)
Solvatomorph A	17,18	–	H_2_O	12.59
	52,79	79	Pyridine	9.32
Solvatomorph B	27,31,41,43,56	56,74	*sec*-Butanol	10.04
Solvatomorph C	28,30,42,44,73	73	DMF	8.89
Solvatomorph V	15,19,27,29,43,45,59	44,45,60	Isopropanol	10.16

※ “–” means that no signal detected

### TG and DTA of the Four Solvatomorphs of BE

**Fig. 1:**
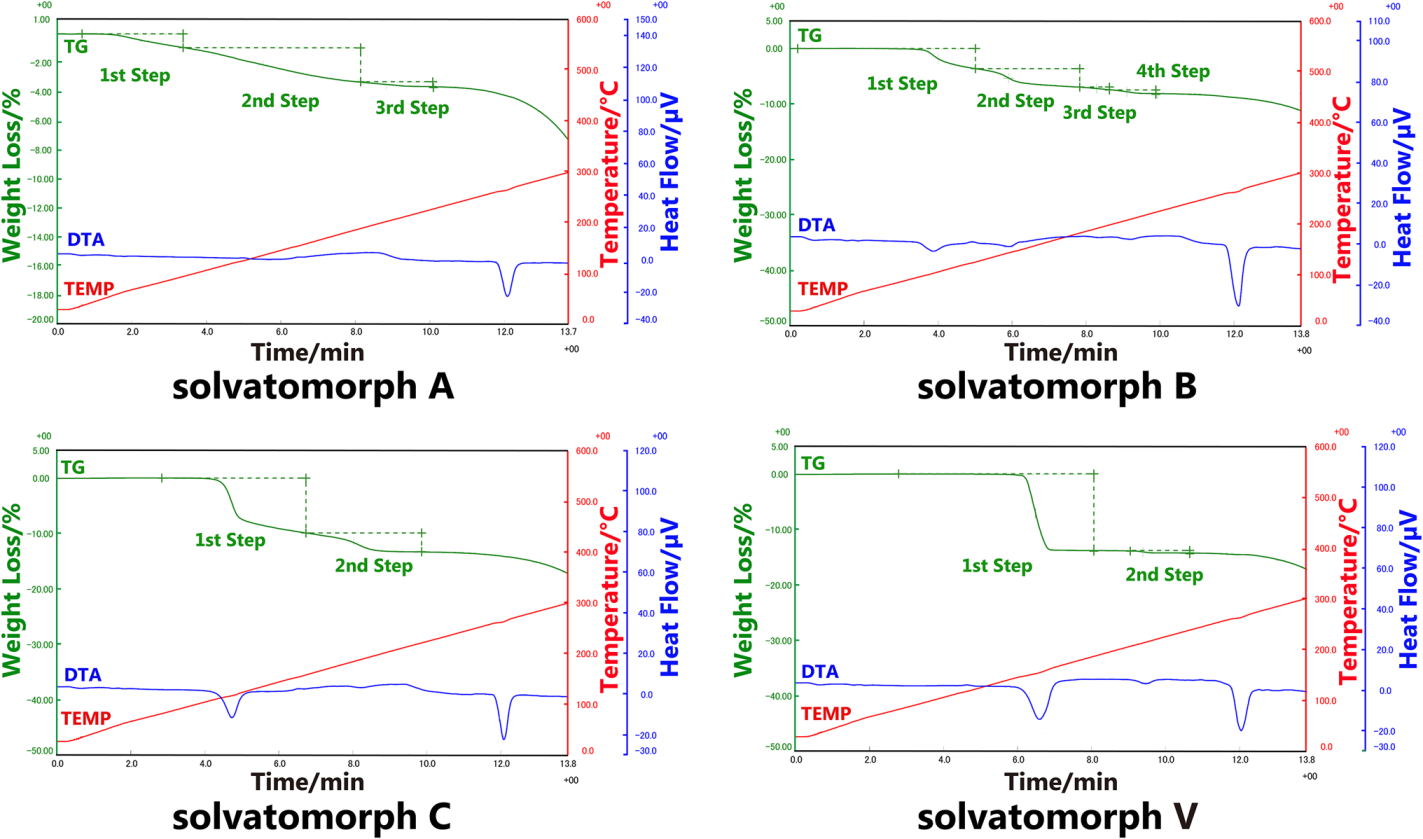
TG, DTA and temperature curves of four solvatomorphs of BE

According to the TG results, all four solvatomorphs exhibited more than one weight loss-step in the range of 30–250 °C, suggesting that the desolvation process, as a characteristic thermal behavior of solvates, can be dispersed in sequence. For the solvatomorphs B, C, and V, the endothermic peaks caused by the desolvation process occurred in their DTA curves. The details of the TG–DTA results are shown in Table 3. The TG and DTA curves of the four solvatomorphs are shown in Fig. 1.

**Table 3:** TG–DTA results of four solvatomorphs

Solvatomorphs	Sample weight(mg) in different method	Weight loss percentage (%) of different step	Peak temperature of corresponding endothermic peaks in DTA (°C)
Solvatomorph A	EI 7.15	1st Step 0.93	–
	PI 4.47	2nd Step 2.39	–
		3rd Step 0.31	–
Solvatomorph B	EI 8.02	1st Step 3.62	103.1
		2nd Step 3.35	143.4
	PI 11.13	3rd Step 0.46	–
		4th Step 0.70	–
Solvatomorph C	EI 5.79	1st Step 9.91	117.7
	PI 7.94	2nd Step 3.44	–
Solvatomorph V	EI 6.30	1st Step 13.79	155.0
	PI 8.96	2nd Step 0.45	215.0

※ “–” means that corresponding endothermic peaks in DTA curves was not obvious

### Desolvation and Mass Spectrometry Characteristics of the Four Solvatomorphs of BE

**Fig. 2:**
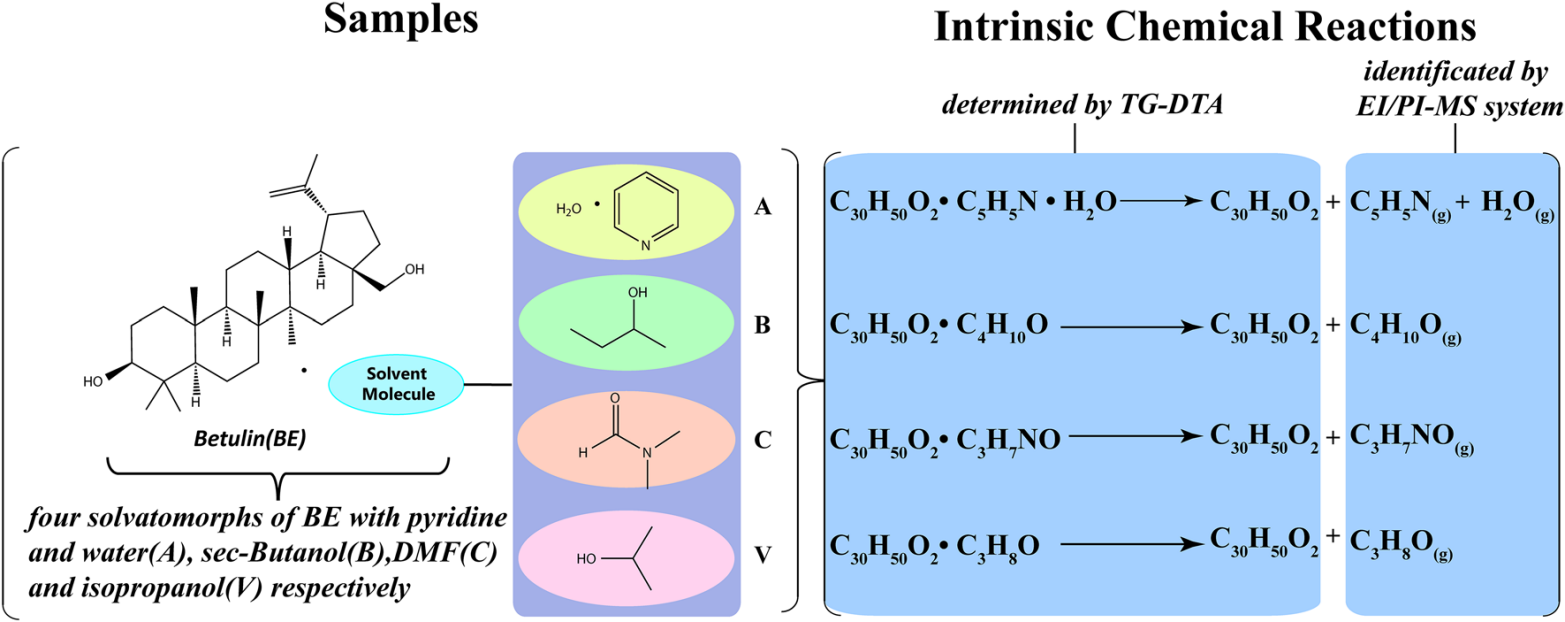
Mechanism of analysis on the four solvatomorphs of BE by the TG–DTA–EI/PI–MS system Equipped with the skimmer-type interface

The weight losses of the solvatomorphs and the ingredients of their respective evolved volatile gases could be determined clearly owing to the real-time and onsite measurements of the TG–DTA–EI/PI–MS system. Figure 2 shows the mechanism of analysis of the four solvatomorphs of BE. Desolvation behavior, as a kind of decomposition reaction, commonly manifested in the solvatomorphs. The distinctions of characteristics of the desolvation processes and mass spectrometry can be attributed to the different solvatomorphs.

#### Solvatomorph A

**Fig. 3:**
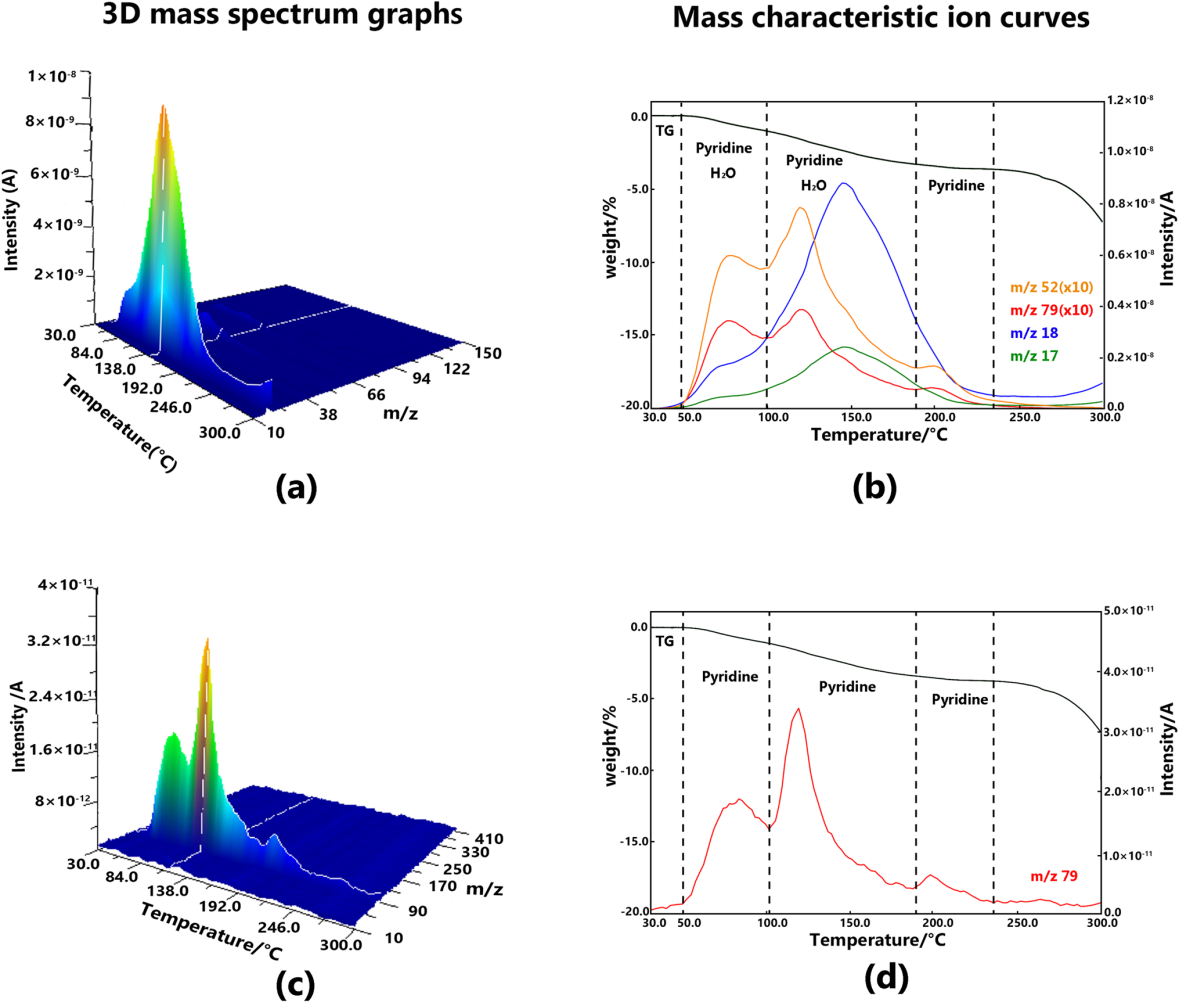
3D mass spectrum graphs of solvatomorph A measured at EI method (**a**) and PI method (**c**), mass characteristic ion curves solvatomorph A measured at EI method (**b**) and PI method (d) ※ “(× 10)” means that the signal intensity was magnified ten times

Three steps of the weight loss were observed in the TG curve. The losses were due to the release of evolved gases consisting of solvent molecules in the solvatomorph. According to the corresponding mass characteristic ion curves, the first and second weight losses around 80 °C were caused by the evolution of pyridine and H_2_O. However, the third weight loss around 200 °C was caused mainly by the evolution of pyridine. Besides, the evolution of H_2_O lagged behind the evolution of pyridine. The molecular ions of pyridine at m/z 79 was remarkable based on the PI measurements. Additionally, as depicted by the TG curve, the speed of the desolvation process was relatively slow, and the temperature span of the whole desolvation process was from 50 to 200 °C. The 3D mass spectrum graph and the mass characteristic ion curves of solvatomorph A measured by the EI and PI methods are shown in Fig. 3.

#### Solvatomorph B

**Fig. 4:**
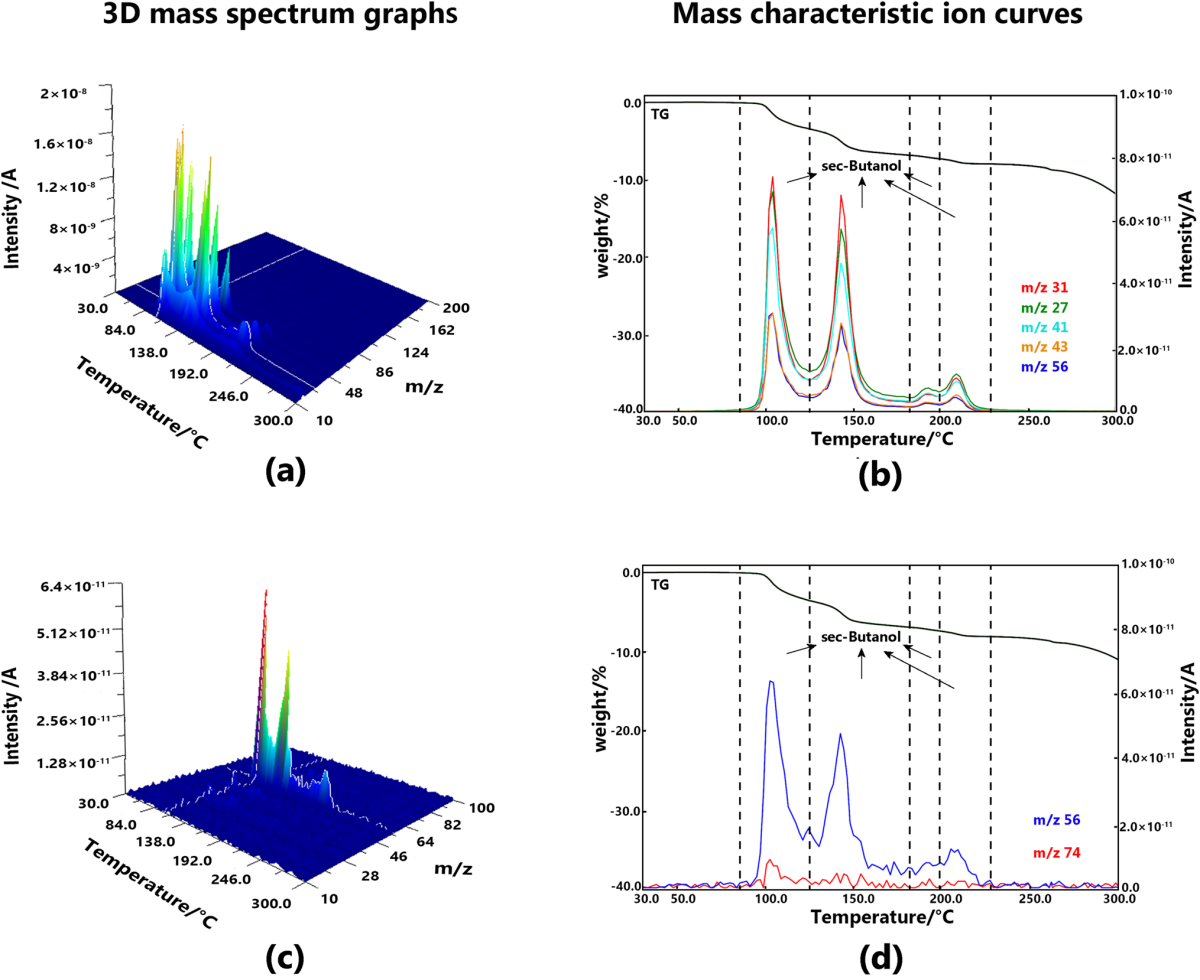
3D mass spectrum graphs of solvatomorph B measured at EI method (**a**) and PI method (**c**), mass characteristic ion curves solvatomorph B measured at EI method (**b**) and PI method (**d**)

Four steps of the weight loss were observed in the TG curve. All these losses were caused by the evolution of *sec*-butanol in reference to the MS analysis results. Notably, in the PI measurement method, the *sec*-butanol molecules were fragmented further to generate new ones (C_4_H_8_^+^, m/z 56), and the signal intensity of the butylene fragments was much higher than that of the *sec*-butanol fragments (C_4_H_10_O^+^, m/z 74). However, the assignment of characteristic peaks remained obvious and relatively simple. The desolvation process of solvatomorph B was fairly gentle, and it occurred between 100 and 200 °C. Figure 4 shows the 3D mass spectrum graph and the mass characteristic ion curves of solvatomorph B.

#### Solvatomorph C

**Fig. 5:**
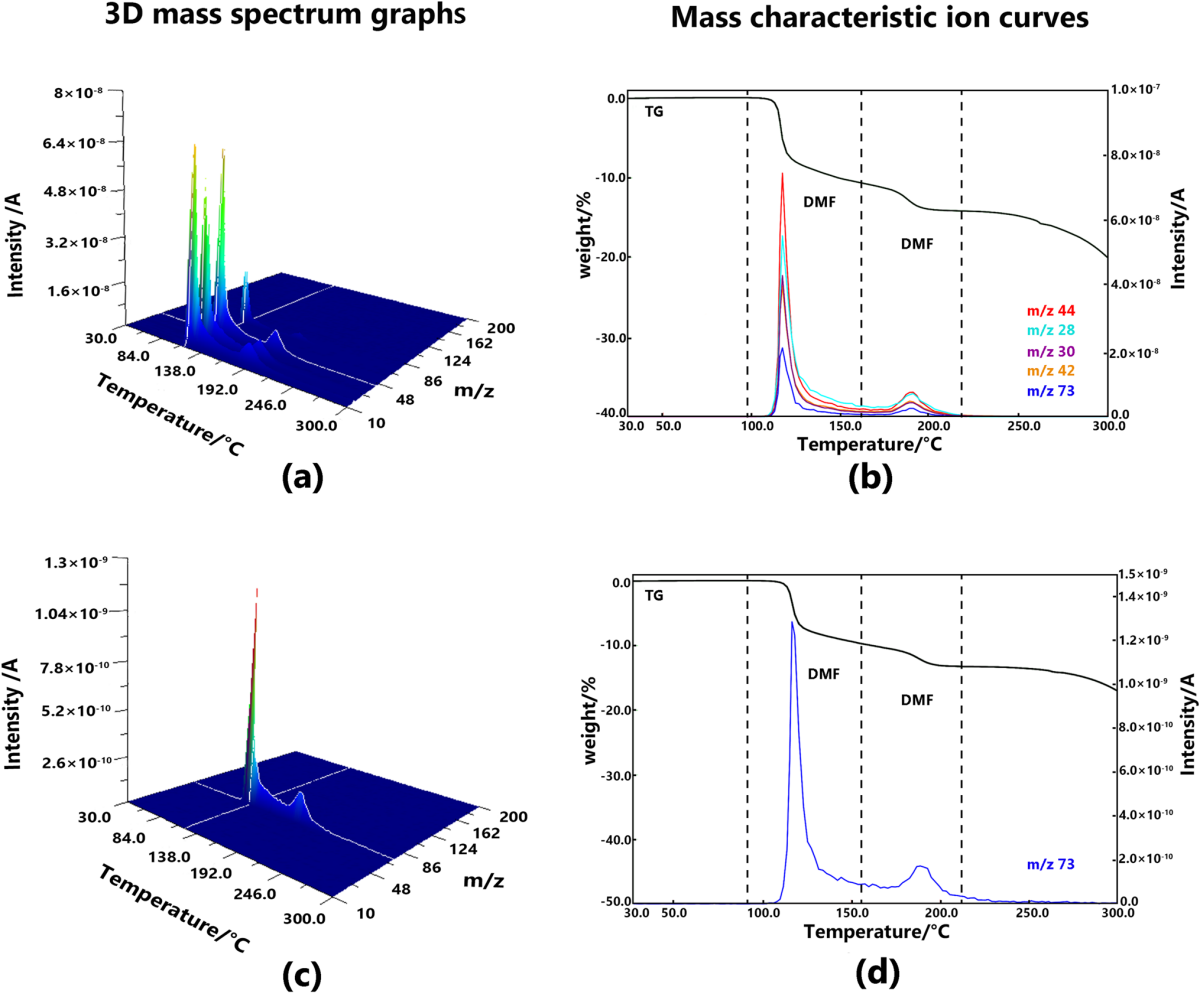
3D mass spectrum graphs of solvatomorph C measured at EI method (**a**) and PI method (**c**), mass characteristic ion curves solvatomorph C measured at EI method (**b**) and PI method (**d**)

Two steps of the weight loss were observed in the TG curve, and both were caused by the evolution of DMF. Only a single fragment of the molecular ions (C_3_H_7_NO^+^) of DMF at m/z 73 was observed in the PI measurements. However, the DMF molecule generated many fragments in the EI measurements. The difference in the slopes of the two weight-loss steps suggests that the first desolvation process is more rapid than the second desolvation process. The complete desolvation process occurred in the range between 80 and 200 °C. Figure 5 shows the 3D mass spectrum graph and the mass characteristic ion curves of solvatomorph C.

#### Solvatomorph V

**Fig. 6:**
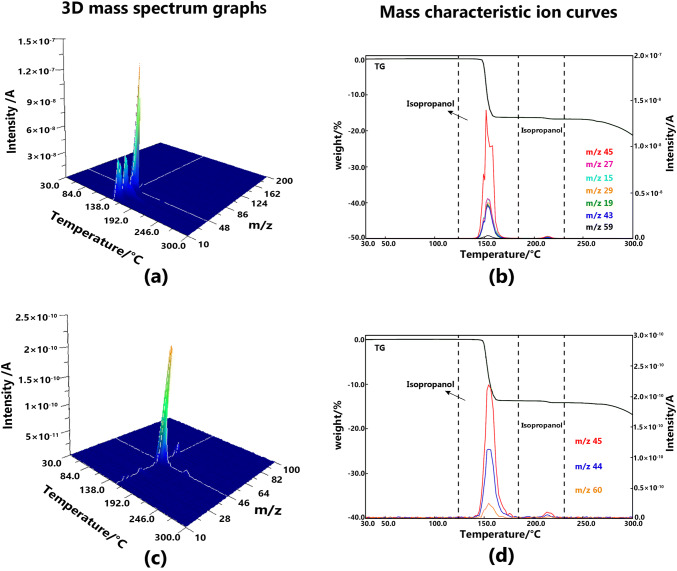
3D mass spectrum graphs of solvatomorph V measured at EI method (**a**) and PI method (**c**), mass characteristic ion curves solvatomorph V measured at EI method (**b**) and PI method (**d**)

The evolution of isopropanol led to two weight-loss steps. After the first large weight loss around 150 °C, an unusual small weight loss occurred around 220 °C. These two weight loss steps were both steep and distinct. For solvatomorph V, the ending temperature of the desolvation process should be considered with the position of the second weight loss, which was close to the melting point of BE. With regard to the MS results, several fragments in the EI measurement method caused challenges in the assignment of characteristic peaks, whereas the fragments decreased noticeably in the PI measurement method. C_2_H_5_O^+^ and C_2_H_4_O^+^, which are the fragments from isopropanol, could be identified easily. The 3D mass spectrum graph and the mass characteristic ion curves of solvatomorph V are shown in Fig. 6.

### Discussion on the Relationship Between Desolvation Behavior and Intermolecular Interaction for the Solvatomorphs of BE

The desolvation behaviors of different natural products were distinct despite of the solvates contained by the same solvents. The desolvation behaviors, as a characteristic thermodynamics property of solvates, were closely related to the intermolecular interactions between the host molecules and the guest molecules in the solvatomorphs. On the one hand, the intermolecular interactions between the host molecules and the guest molecules were much weaker than those in the covalent bonds, and this phenomenon caused the desolvation process to occur in the low temperature range in the TG measurement. On the other hand, the occurrence of intermolecular interactions between the solvent molecules and the host molecules resulted in the practical temperature of the desolvation process, which differed from the boiling point of the corresponding pure solvent.

**Fig. 7:**
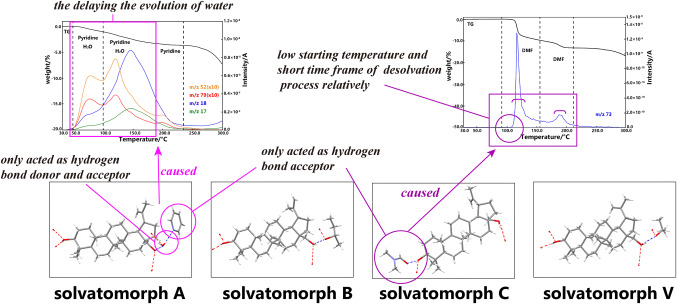
Demonstration of discussion on the relationships between the desolvation behavior and intermolecular interactions in solvatomorphs of BE (the stick models of the single crystals of four solvates are in the bottom, the red and blue dotted lines represent hydrogen bonds interactions between different molecules)

With regard to the four solvatomorphs in this study, according to a previous research on the single-crystal structural analysis of these four solvatomorphs of BE [8, 9], the main intermolecular interactions existing between the solvent molecules and the BE molecules were all classic hydrogen bond interactions via O–H⋯O. Notably, only the DMF molecules in C acted merely as a hydrogen bond acceptor, whereas other solvent molecules, such as the water in A, *sec*-butanol in B, and isopropanol in V, acted as both a hydrogen bond donor and an acceptor. The pyridine molecules in A only acted as hydrogen bond acceptor, and no hydrogen bond interactions existed between the pyridine and BE molecules due to the water molecules relayed between these two elements. This finding may explain the phenomenon that the evolution of H_2_O, which was slower than the evolution of pyridine during the measurements. Besides, the positions of the hydrogen bond interactions were the same in the BE molecules for solvatomorphs A, B, and V. The position of the hydrogen bond interactions between the DMF molecules and BE molecules in C was distinct obviously from the other three solvates of BE. The above results on crystal structural analysis have been verified by a previous research that utilized Hirshfeld surface analysis. This characteristic of the crystal structure of C may explain the evolution process of DMF in C, which started at the low temperature, and was also relatively quick, because of the weak intermolecular interactions in C. Meanwhile, the desolvation processes of A and B continued at a much longer time, whereas the desolvation process of V began at a higher temperature given the same measurements conditions. Figure 7. demonstrates the discussion presented above.

### Comparison of EI and PI Method on Analyzing Solvatomorphs of Natural Products

The two methods both have advantages and disadvantages based on the analyses of the solvatomorphs of the natural products.

For the EI method, the MS signal intensity was much higher than that in the PI method because of its higher ionization energy. The EI method is suitable for all kinds of evolved organic volatile gases, even the various gaseous organic or inorganic matters. However, the overlay of characteristic peaks caused by the excessive fragments is inevitable, and this phenomenon will lead to challenges in the assignment of characteristic peaks.

The PI method, as a soft ionization method, can provide more accurate information on evolved volatile gases by means of allowing the molecules to remain intact as much as possible. For solvatomorphs A and C in this study, pyridine and DMF were detected on the basis of their molecular ions, and no other fragments appeared in the measurement. However, the application range is limited to a certain extent because of its low ionization energy. For instance, H_2_O in solvatomorph A cannot be detected by the PI method because the ionization energy of H_2_O molecules is higher than 10.2 eV. According to the ionization energy from the NIST Library Database [19], the solvents theoretically suitable for detection by the PI method included the four solvents identified in this study (pyridine, *sec*-butanol, DMF, and isopropanol) and 15 other organic solvents (benzene, cyclohexane, 1,4-dioxane, 1,2-ethanediol, n-hexane, 2-methoxyethanol, tetrahydrofuran, toluene, acetone, 1-butanol, dimethyl sulfoxide, ethyl acetate, ethyl ether, 2-butanone, and xylene).

However, the combination of the two ionization methods can complement each other greatly. In analyzing the solvatomorphs of natural products, we recommend the selection of an appropriate ionization method in concordance with the solvents possibly contained by the solvatomorphs. As for completely unknown simples, the EI method should be initially employed. Afterwards, the PI method can be considered as a means of further verifying the assignment results of the characteristic peaks in the EI measurements.

## Conclusions

All four solvatomorphs of BE were identified and characterized distinctly by the skimmer-type interfaced TG–DTA–EI/PI–MS system. Their respective weight losses in the TG curves aligned well with the characteristic fragment curves in MS. Their thermodynamic reaction processes were also explored by this system. The following conclusions were demonstrated:

1. Owing to the introduction of the PI method, the assignment process of the characteristic peaks was greatly simplified compared with the process involved in the standard EI method. For the four solvatomorphs of BE, the five kinds of the main volatile gaseous species, which consisted of H_2_O, C_5_H_5_N (pyridine) from A, C_4_H_10_O (*sec*-butanol) from B, C_3_H_7_NO (DMF) from C, and C_3_H_8_O (isopropanol) from D, were identified distinctly from the EI–MS and PI-MS measurements.
2. Through this system, the multi-step desolvation process of all the four solvatomorphs of BE was revealed. The differences in the reaction speeds and temperatures of their respective desolvation processes were related to the intermolecular interactions between the BE molecules and the solvent molecules. However, the ending temperature of the desolvation processes of all the four BE solvatomorphs exceeded 200 °C, nearing the melting point of BE, which suggests that the desolvation temperature of BE should be considered carefully during the purification and preparation, and even the quality control process of BE.
3. In analyzing the different solvatomorphs of the natural products, the EI method and the PI method should be purposefully employed in combination to achieve a simple and fast analysis process.

In the foreseeable future, we have a reason to believe that the TG–MS technique will become a powerful and superior technique for rapid screening, as it can identify and characterize the various solvatomorphs of natural products.

## Experiment Section

### Materials

The raw material powder of BE was purchased from Nanjing Zelang Pharmaceutical Technology Co., Ltd. (Nanjing, China, Batch No.: 20140528). The chemical purity of this powder was higher than 99%, as determined by high-performance liquid chromatography. All solvents used for crystallization were of analytical grade and purchased from Beijing Chemical Works (Beijing, China).

### Preparation of Samples

Approximately 200 mg of the BE raw material was added to 30 mL of different solvents, namely, pyridine, *sec*-butanol, DMF and isopropanol. The solutions were stirred for 12 h in ambient temperature and then filtered to obtain clear and saturated solutions. The solutions were allowed to stand for crystallization for about half a month at 10 °C to obtain single crystals of solvatomorphs. Small pieces of the single crystals of the four solvatomorphs of BE were ground to powder. The obtained powder was sifted by a 100-mesh sieve to meet the requirements of the TG–DTA–EI/PI–MS measurements.

### Instrumentation

**Fig. 8:**
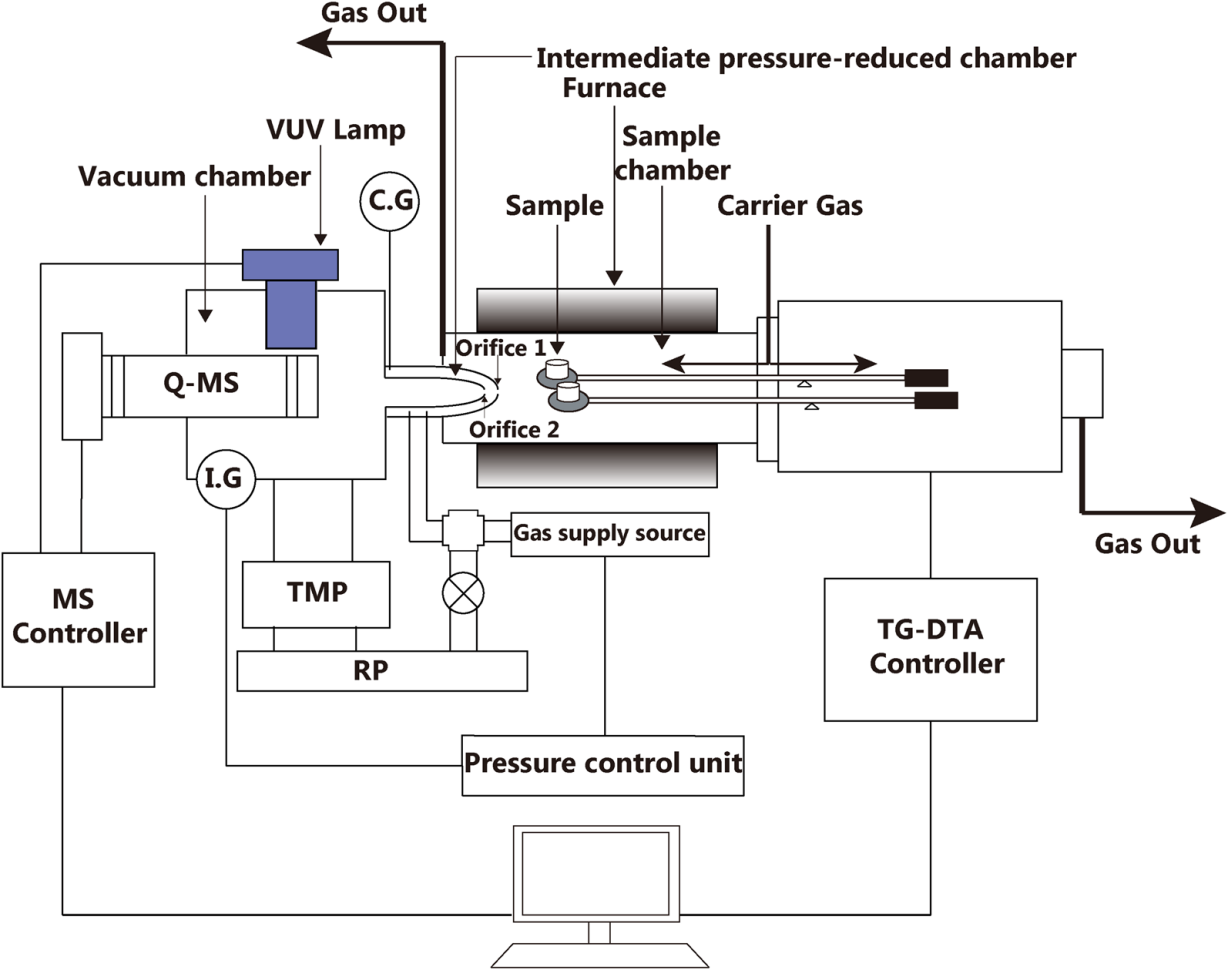
Schematic diagram of TG–DTA–EI/PIMS system using a skimmer type interface. (*IG* ion gauge, *CG* crystal gauge, *TMP* turbo molecular pump, *RP* Rotary pump)

The TG–DTA–EI/PI–MS measurements were performed on the ThermoMass Photo/H (Rigaku Corporation, Tokyo, Japan). Figure 8 shows the schematic diagram of the TG–DTA–EI/PI–MS system. The cylindrical quadrupole MS and the horizontal TG–DTA comprised the two main parts of the system. A skimmer-type gas injection interface was fitted between the MS and the TG–DTA to connect the two parts. Two concentric quartz tubes with orifices connected both devices in the skimmer-type interface. The length of the interface was set to be short as possible. Considering the effects of MS at high vacuum and the sample chamber (TG–DTA) at a certain atmospheric pressure, an intermediate pressure-reduced atmosphere evacuated by the rotary pump (RP), existed between the outer skimmer and the inner skimmer. In addition, with regard to the MS ion source, the two orifices of the skimmer-type interface and the sample in the TG–DTA were adjoined to each other and arranged coaxially. Consequently, the onsite and real-time measurements of the samples could be achieved successfully. At the same time, the re-condensation of the evolved gases could be avoided remarkably. More details on the TG–DTA–EI/PI–MS system can be read from the literature [20–22].

The ionization of the evolved volatile gases was fulfilled by two methods, namely, the standard EI at 70 eV and the PI irradiated by a vacuum ultraviolet (VUV) deuterium discharge lamp at λ = 121.6 nm and hv = 10.2 eV. The position of the VUV of the PI source was close to the EI ion source. Different from the EI method, the PI method, as a soft ionization technique, can provide direct information on evolved volatile gases owing to the much fewer fragments produced during measurement. For example, the DMF molecule can be simply ionized as C_3_H_7_NO^+^ under the low photon energy of 10.2 eV in the PI–MS measurement; by contrast, it will be bombarded with an electron beam of 70 eV kinetic energy to produce excessive fragments in the EI–MS measurement. Nevertheless, the PI method is not suitable for certain evolved volatile gases, such as methanol, or H_2_O, whose molecules require much higher ionization energy.

### Experimental Conditions

All samples were weighed in Al crucibles and heated to 300 °C with a controlled temperature program. All measurements were performed at the heating rate of 20 °C·min^−1^ under high purity analytical grade dry He gas with a flow rate of 150 mL·min^−1^. All samples were measured by Scan Mode (m/z 10–410), with sampling performed every second. The detailed information of the samples used in the measurements are shown in Table 3.
